# Two-Player Game in a Complex Landscape: 26S Proteasome, PKA, and Intracellular Calcium Concentration Modulate Mammalian Sperm Capacitation by Creating an Integrated Dialogue—A Computational Analysis

**DOI:** 10.3390/ijms21176256

**Published:** 2020-08-29

**Authors:** Angela Taraschi, Costanza Cimini, Giulia Capacchietti, Marina Ramal-Sanchez, Luca Valbonetti, Juliana Machado-Simoes, Fadl Moussa, Israiel Tagaram, Samia Mokh, Mohamad Al Iskandarani, Alessia Colosimo, Barbara Barboni, Nicola Bernabò

**Affiliations:** 1 Faculty of Bioscience and Technology for Food, Agriculture and Environment, University of Teramo, 64100 Teramo, Italy; ataraschi@unite.it (A.T.); ccimini@unite.it (C.C.); gcapacchietti@unite.it (G.C.); mramalsanchez@unite.it (M.R.-S.); lvalbonetti@unite.it (L.V.); jsomoesmachado@unite.it (J.M.-S.); fmoussa@unite.it (F.M.); itagaram@unite.it (I.T.); acolosimo@unite.it (A.C.); bbarboni@unite.it (B.B.); 2 Istituto Zooprofilattico Sperimentale dell’Abruzzo e del Molise “G. Caporale”, Via Campo Boario 1, 64100 Teramo, Italy; 3 National Research Council—Institute of Biochemistry and Cell Biology (IBBC), A. Buzzati-Traverso Campus, via E. Ramarini 32, I-00015 Monterotondo Scalo, Roma, Italy; 4 Doctoral School of Science and Technology Lebanese University, Beirut 1107-2260, Lebanon; 5 National Council for Scientific Research (CNRS), Lebanese Atomic Energy Commission (LAEC), Laboratory for Analysis of Organic Compound (LACO), Beirut 1107-2260, Lebanon; s.mokh@laec-cnrs.gov.lb; 6 Faculty of Public Health I—Lebanese University, Hadath 1107-2260, Lebanon; mohamada@ul.edu.lb

**Keywords:** male gametes, sperm capacitation, protein phosphorylation, biological network, computational modeling, sperm signaling

## Abstract

Recent experimental findings suggest the involvement of the 26S proteasome, the main protease active in eukaryotic cells, in the process that leads mammalian sperm to become fully fertile, so-called capacitation. Unfortunately, its role in male gametes signaling is still far from being completely understood. For this reason, here, we realized a computational model, based on network theory, with the aim of rebuilding and exploring its signaling cascade. As a result, we found that the 26S proteasome is part of a signal transduction system that recognizes the bicarbonate ion as an input terminal and two intermediate layers of information processing. The first is under the control of the 26S proteasome and protein kinase A (PKA), which are strongly interconnected, while the latter depends on intracellular calcium concentrations. Both are active in modulating sperm function by influencing the protein phosphorylation pattern and then controlling several key events in sperm capacitation, such as membrane and cytoskeleton remodeling. Then, we found different clusters of molecules possibly involved in this pathway and connecting it to the immune system. In conclusion, this work adds a piece to the puzzle of protease and kinase crosstalk involved in the physiology of sperm cells.

## 1. Introduction

Mammalian spermatozoa are infertile immediately after ejaculation and need to undergo functional maturation to acquire the competence to fertilize the female egg. This process, known as capacitation, requires typically from hours to days (depending on the species), and is completed in vivo in the sperm reservoir, i.e., the reservoir formed by the spermatozoa when they bind to the epithelium of the oviductal cells prior to fertilization, concretely in the isthmic portion of the oviduct [1]. In this organ, a complex network of interactions involving the oviductal epithelial cells and the tubal fluid (whose composition depends on the neuroendocrine axis of female) drives the functional maturation of the male gametes, thanks to a well-balanced action of inhibiting and activating molecular messages. Capacitation leads to an increase in sperm plasma membrane fluidity due to cholesterol loss [2,3], an increase in ionic influx by membrane hyperpolarization [4], and important changes in the protein phosphorylation pattern [5].

In this context, the role of the 26S proteasome is emerging, i.e., the main protease in eukaryotic cells [6], formed by a multi-enzymatic complex with trypsin-like, chymotrypsin-like, and peptidylglutamyl peptide-hydrolyzing (PGPH) activities [7]. It is composed of two subcomplexes: the 20S core, which is the proteolytic component, and the 19S regulatory particle, responsible for recognizing, unfolding, and translocating the ubiquitinated substrates into the 20S core [8]. The 20S core is made up of four stacked rings, two α and two β, each made of seven subunits, with the β rings containing the proteolytic enzymes [6]. The 19S regulatory particle consists of a base and a lid and forms a complex that binds an additional subunit, Rpn10 [6]. The substrate proteins to the 26S proteasome are modified with ubiquitin chains [9] and directed into the 20S core where they are degraded, whereas most of the ubiquitin molecules are recycled [8]. They use the hydrolysis of ATP molecules to modify complex substrate structures, thus modulating the cellular proteome and degrading numerous regulatory proteins and damaged or misfolded polypeptides [10]. Their role was demonstrated in many biologic processes, such as immune and inflammatory responses, morphogenesis of the neuronal network, cell cycle, modulation of cell surface receptors [11], and reproduction [12]. In particular, in human spermatozoa, it is located on the plasma membrane overlying the sperm acrosome, underside the plasma membrane, in the acrosomal matrix, in the inner acrosomal membrane, and finally in the sperm tail [12].

Currently, a huge amount of molecular data concerning sperm capacitation in several mammalian models are available, due to the large-scale adoption of the so-called –omics (proteomics, lipidomics, transcriptomics, etc.) and, thus, the involvement of the 26S proteasome in this context was proposed. Unfortunately, these molecular data are still unorganized, and the 26S proteasome’s role in the flux of information that drives sperm physiology is still unclear. For this reason, here we realized a computational model, based on the application of the network theory, as an attempt to better understand this important issue.

We adopted this strategy because we know that sperm capacitation is a complex system, as all other signaling pathways. In other words, it is characterized by the interaction of several entities (molecules) that define a specific pattern; it has feedbacks and loops, the interactions are often non-linear, the response to external or internal stimuli is non-proportional to the stimulus intensity. and the system has a memory of prior states. In this light, the modeling of the system as a network represents a good option for studying its complexity; indeed, it allows taking into account the molecular components of the system itself, as well as their interactions, in keeping with the classical sentence claiming that “in complex systems, a system is more (different) than the sum of its parts”.

To this aim, here, we focused our model on studying the role of the 26S proteasome in mammalian sperm capacitation, aggregating all the molecular data available to date and realizing the network that represents its interactome (26SN).

## 2. Results and Discussion

We obtained a network (26SN) whose most relevant topological parameters are listed in Table 1, while further information about the single-node topology is provided in Table S1.

Despite the relatively small number of nodes, based on the parameters listed in Table 1, it is possible to classify the network as adherent to the Barabasi–Albert model (BA) of scale-free networks [13]. Indeed, the presence of hyper-connected nodes (hubs) coexists with the absence of correlation between node degree and clustering coefficient (*R^2^* = 0.385), whose value is near zero (0.016).

Thus, we explored the controllers active within the network, searching for the most connected nodes, i.e., the hubs. As a result, we found that the hubs within our network are the 26S proteasome, PKA, and Ca^2+^. Interestingly, we found that they are also the controllers of information flow, as demonstrated by their higher value of bottleneck score (see Table S2).

**Figure 1:**
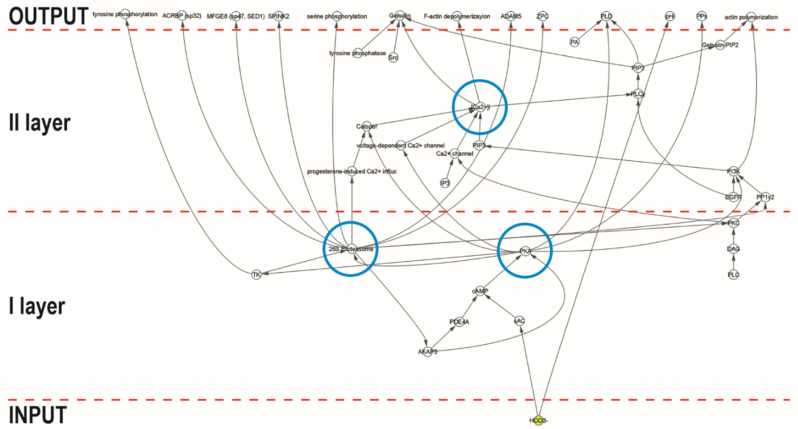
Hierarchical layout of 26SN. Representation of the network topology using a hierarchical layout that organizes the network depending on the direction of links. The blue circles indicate the controllers of information flux, which are implied in different layers of the signaling system.

To better define their role in the context of network topology, we represented them using a hierarchical layout that organizes the network depending on the direction of links, thus showing the information or mass flux within the network itself (Figure 1). Indeed, using this layout, the “nodes are placed in hierarchically arranged layers, and the ordering of the nodes within each layer is chosen in such a way that minimizes the number of edge crossings” (Cytoscape Manual http://manual.cytoscape.org).

In this way, it emerges that the functional role exerted by these nodes is different and refers to their location in different layers of the signaling system; the pathway starts with the ion HCO_3_^−^, which acts as an activating factor of the cAMP/PKA cascade. Then, it is possible to identify a first layer of the signaling system, characterized by the strong cross-link between the 26S proteasome and PKA. In this context, we know that, during capacitation, HCO_3_^−^ influx activates the sperm protein soluble adenylyl cyclase (sAC), increasing the intracellular pH [14]. The activation of sAC results in increased levels of cAMP and PKA activity. The latter is cAMP-dependent and is restricted to a discrete location within the sperm, binding A-kinase anchoring proteins (AKAPs) [4]. Indeed, PKA enzyme can bind AKAPs, mostly AKAP-3 and -4, to the regulatory subunit of the protein. AKAP-4 is the major component of sperm fibrous sheaths and possesses the ability to form molecular complexes with other signaling proteins. Its amphipathic helix constitutes a specific PKA-binding domain, participating in this way in the regulation of PKA. The binding of cAMP to the PKA regulatory subunits allows the dissociation of the tetramer and the activation of the catalytic subunit, initiating a cascade of intracytoplasmic signaling events [15]. Once free, the catalytic subunits remain active to phosphorylate a wide variety of substrates on Ser/Thr residues, directly or indirectly activating diverse protein kinases and/or inhibiting protein phosphatases, producing an increase in the phosphorylation of tyrosine residues [3,5] and contributing to reshaping the global protein phosphorylation pattern. This change was demonstrated to occur either in the flagellum or in the sperm head, and it appears to be a necessary prerequisite for a spermatozoon to reach the ability to fertilize the oocyte [3,5]. PKA is responsible for the phosphorylation of multiple subunits of the 26S proteasome, thus increasing its enzymatic activity [14]. This phosphorylation is essential for the assembly of the regulator complex, 19S, with the 20S complex [16]. In addition, the chymotrypsin-like and trypsin-like activities of the proteasome are directly stimulated by phosphorylation of Ser120 of the Rpt6 subunit [17].

In turn, the proteasome is involved in intracellular signaling pathways in the second layer of information processing, which leads to an increase in the degree of sperm protein phosphorylation. It directly or indirectly modulates the Ser and Thr phosphorylation, degrading serine phosphatases, threonine kinases, or proteins phosphorylated at Thr residues during capacitation [16]. A target of the proteasomal degradation is AKAP3. This protein anchors PKA in the sperm tail, limiting its localization in this compartment, where its activity is required for the phosphorylation of proteins involved in motility [18]. Under capacitation conditions, the dissociation between PKA and AKAP3 and the intracellular alkalization enhance the AKAP3 degradation via proteasomal machinery. The AKAP dissociation and degradation enable a shift of PKA from the tail to the head to promote the acrosome reaction [18].

The proteasomal activity is also involved in the redistribution and turnover of sperm surface protein implicated in the formation of the sperm-oviductal epithelium reservoir (MFGE8, ADAM5, AQN1), sperm-ZP binding (MFGE8, AQN1), and sperm plasma membrane fusion with the oolema (ADAM5) [19,20,21]. Thus, the involvement of the sperm proteasome with acrosomal function starts as early as sperm capacitation and may influence acrosomal remodeling, sperm detachment from the oviductal sperm reservoir, and the sperm–egg interaction [21]. Finally, the sperm proteasome may be involved in the exocytosis of the acrosome, in events upstream of the plateau phase of the Ca^2+^ influx during the acrosome reaction. This may decrease the sustained phase of Ca^2+^ influx, without affecting the initial transient peak [9].

The second layer of signaling systems is under the control of Ca^2+^. The intracellular calcium homeostasis is one of the key parameters that drives sperm function. In resting conditions, the calcium clearance is maintained by molecules acting either at the plasma membrane or at the cytosolic level. The plasma membrane Ca^2+^-ATPase and Na^+^/Ca^2+^ exchanger export Ca^2+^ ions from the cytoplasm to the extracellular environment [4]. At the cytosolic level, the sarcoplasmic–endoplasmic reticulum Ca^2+^-ATPase pumps and the mitochondrial Ca^2+^ uniporter operate sequestering calcium into the acrosome or into the mitochondria, respectively [22]. During capacitation, the intracytoplasmic calcium levels increase as a consequence of the Ca^2+^ influx from the extracellular environment and the Ca^2+^ efflux from the intracellular stores located in the acrosome and at the redundant nuclear envelope (RNE). In detail, voltage-dependent calcium channels (VDCCs), transient receptor potential channels (TRP channels), cyclic nucleotidic gated channels (CNG channels), and CatSper channels are activated, promoting the increased of intracytoplasmic Ca^2+^ concentration [23]. CatSper channels have a key role in the acquisition of hyperactivated motility and are located on the principal piece of the sperm flagellum [24]. They are strongly potentiated by alkaline pH, but they also respond to other physiological stimuli, such as progesterone [4]. Sperm from CatSper-null mice are motile, but sterile because they fail to hyperactivate and cannot fully ascend the female genital tract or penetrate the zona pellucida [24]. Moreover, they were recently shown to be necessary for the sperm detachment from the oviductal reservoir in swine [25]. Sperm intracellular Ca^2+^ can be released from the acrosome and RNE by inositol triphosphate and ryanodine receptors—IP3R and RyR, respectively [4,23].

As a final consequence of the pathway activation, the terminal events in signaling are stimulated, both at the membrane and at the cytoskeleton level. In this context, it is worth reminding that spermatozoa are unable to synthesize new proteins and that their cytosol is virtually absent. Therefore, their signaling system is mainly located in the membrane and in the actin cytoskeleton. Both are very dynamical structures, whose architecture changes depend on the balancing of activating or inhibiting stimuli. The result of such an evolution will be the definition of the final fate for the male germ cells: the acquisition of fertilizing ability or its destruction (for instance, by activating pro-apoptotic pathways). The membrane lipid scrambling is activated; the sperm head plasma membrane (PM) has two leaflets, inner and outer ones, which differ in their composition, and this asymmetry is established and maintained by several translocating enzymes with differing phospholipid specificities [2,26,27]. During capacitation, this phospholipid asymmetry is reduced by scramblase, which acts as a bi-directional carrier with little specificity, moving phospholipids in both directions (inward and outward) according to their concentration gradient [2]. As a result of the LR, sperm membranes undergo a deep rearrangement that affects their composition and their biophysical properties, increasing their fluidity and fusogenicity [28]. This process enables the extracellular protein to extract cholesterol, determining the increase in the ability of the sperm plasma membrane (PM) and the outer acrosome membrane (OAM) to fuse (fusogenicity), which is a necessary prerequisite for the acrosome reaction once the zona pellucida of the oocyte is met [2].

Membrane reorganization is deeply related to the remodeling of the actin cytoskeleton, whose changes are regulated by the crosstalk between protein kinases A (PKA) and C (PKC) and the activation of phosphatidylinositol 3-kinase (PI3K). The PI3K activation leads to an increase in actin polymerization; thus, the G-actin polymerizes, forming long filaments of F-actin [3]. The F-actin constitutes a barrier for the fusion between the OAM and PM and, when the capacitation progresses, the interposition of F-actin avoids their premature fusion [28,29]. In response to the physiological stimulus represented by contact with proteins of the oocyte ZP, high intracellular calcium concentrations enable the depolymerization of actin, since F-actin breakdown between the membranes is needed to allow the acrosome reaction [28].

As spermatozoa bind to ZP and undergo acrosomal exocytosis, proteasomes are able to degrade some of the ZP proteins [12]. In details, acrosomal exocytosis results in the exposure of proteasomes associated with the inner acrosomal membrane, where they may provide a sustained proteolytic activity while the sperm head advances through the ZP [12,30].

The model clearly shows as the 26S proteasome complex is active in sperm signaling. It is involved in all steps of the reproductive process, including sperm–zona pellucida interactions [19] and the protein phosphorylation occurring during capacitation [7], contributing to the release of capacitated spermatozoa from the oviductal sperm reservoir [12].

In addition, we carried out an enrichment analysis using STRING, a database that includes known and predicted protein interactions. With this analysis, we obtained a network of molecules that could be possibly involved in 26S proteasome signaling based on the information available derived from different sources (genomic context, high-throughput experiments, conserved co-expression, and previous knowledge), thus identifying new possible players of the 26S proteasome pathway involved in capacitation.

**Figure 2:**
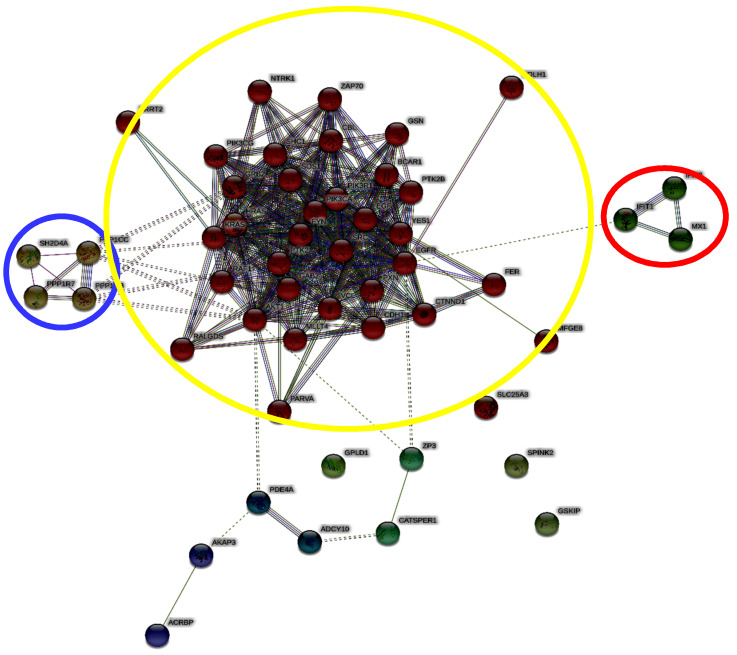
Network representing the enrichment analysis carried out with STRING. The different colors represent different clusters: the main core of interconnected elements (yellow circle); phosphatases (blue circle); and ifit1, ifit3, and mx1, involved in interferon function (red circle).

Then, we used a specific algorithm based on Markov chain (Figure 2) for cluster identification. 

It is very interesting to note that we found a cluster of molecules (yellow circle) that represent the main core of interconnected elements. It contains several protein involved in control of information processing, such as kinases, in cell signal transduction (see Table S3).

Additionally, we found two other clusters. The blue one is constituted by phosphatases (ppp1cc, ppp1r7, ppp1cb, and sh2d4a). It is known that protein phosphorylation is a landmark of sperm capacitation, and it depends upon the balanced action of kinases and phosphatases [5]. These mechanisms of phosphorylation/dephosphorylation are very important in spermatozoa, which are highly specialized cells and transcriptionally silent, except for the mitochondrial protein translation of nuclear-encoded proteins [31].

The second cluster (red one) is constituted by ifit1 and 3 and mx1, which refer to the interferon function. This seems to be a very interesting result and in agreement with our previous study, which showed the “interferon” as a controller of the human sperm interactome, stressing the connection between capacitation and the immune system [32].

As demonstrated here, the adoption of a biological network-based approach allowed us to infer new and interesting processes involved in the functional maturation of male gametes, demonstrating the utility of computational modeling strategies in exploring cell signaling systems. 

In particular, we found that, during capacitation, a pathway constituted by two layers of information processing is active, in which the 26S proteasome, the PKA (first layer), and the intracellular calcium concentration (second layer) play a key role. Then, it was possible to suggest a new possible element active in capacitation, active through protein phosphorylation and connecting it with the immune system.

In this way, the new findings open the way for further studies that could give new information related to sperm physiology and new perspectives in the potential design of diagnostic and therapeutic strategies of great utility for approaching male infertility.

## 3. Materials and Methods

### 3.1. Data Collection, Network Creation, and General Analysis

We found that several online archives contain data regarding the molecular interaction of the 26S proteasome, such as Reactome (reactome.org), KEGG (genomes.jp/KEGG), or Wikipathways (wikipathways.org); however, they always refer to very general contexts, without a specific focus on male gamete physiology. As such, we decided to realize a new database in house. To this aim, we collected the recent scientific literature published in peer-reviewed international papers included in the PubMed archive (http://www.ncbi.nlm.nih.gov/pubmed) [2,33]. Specifically, we used nine papers (PMID: 22427115, 27053366, 23894359, 23001443, 19144957, 15253927, 21383844, 12721178, 31467409) published between 2003 and 2017, focusing on the role of the 26S proteasome in sperm physiology after ejaculation in mammals.

Freely available and diffusible molecules such as H_2_O, CO_2_, Pi, H^+^, and O_2_ were omitted when not necessary, and, in some cases, the record did not represent a single molecule but a complex event, such as “tyrosine phosphorylation”, because all the single-molecular determinants of the phenomenon are still unknown. As a reference, two researchers, experts on spermatozoa biology, carried out an independent literature analysis on papers that referred to the role of the sperm proteasome during capacitation using the same keywords. Then, the databases were compared, and a third researcher verified the correctness of the record inserted, resolving eventual conflicts.

The database (interaction database) was realized in Microsoft Excel 2013 and contained the following fields:

Molecule involved in biochemical reaction (source): molecules participating in the interaction as a source;

Interaction: kind of interaction between the molecules;

Molecule involved in biochemical reaction (target): molecules participating in the interaction as a target;

Alias: eventual aliases;

Role: physiological and/or pathological role of the molecule/reaction related to fertility;

Reference: article reporting the above-mentioned data;

Notes: any further information that could be useful in the study.

Once the database was created, we obtained the 26S proteasome network (26SN).

The proteasome network was realized and analyzed with Cytoscape 3.7.2 and the specific plug-in Network Analyzer. The analysis was carried out considering the network as directed, assessing the topological parameters listed and described in Table 2.

In addition, the betweenness centrality and closeness centrality were computed.

The betweenness centrality *C_b_(n)* of a node *n* is computed as follows:

*C_b_*(*n*) = ∑_*s≠n≠t*_ (*σ_st_* (*n*)/*σ_st_*), (1)

where *s* and *t* are nodes in the network different from *n*, *σ_st_* denotes the number of shortest paths from *s* to *t*, and *σ_st_* (*n*) is the number of shortest paths from *s* to *t* that *n* lies on.

Betweenness centrality is computed only for networks that do not contain multiple edges. The betweenness value for each node *n* is normalized by dividing by the number of node pairs excluding *n*: (*N* − 1)(*N* − 2)*/2*, where *N* is the total number of nodes in the connected component that *n* belongs to. Thus, the betweenness centrality of each node is a number between 0 and 1.

The closeness centrality *C_c_(n)* of a node *n* is defined as the reciprocal of the average shortest path length and is computed as follows:

*C_c_*(*n*) = *1*/*avg*( *L*(*n*,*m*) ), (2)

where *L*(*n*,*m*) is the length of the shortest path between two nodes *n* and *m*. The closeness centrality of each node is a number between 0 and 1.

NetworkAnalyzer computes the closeness centrality of all nodes and plots it against the number of neighbors. The closeness centrality of isolated nodes is equal to 0.

Closeness centrality is a measure of how fast information spreads from a given node to other reachable nodes in the network.

### 3.2. Identification of 26SN Hubs

We identified the hyper-connected nodes, i.e., the hubs, as previously described [2,34], by using the following equation:

(3) ND>μ+σ,

where *ND* is the node degree, *μ* is the mean node degree, and *σ* is the node degree standard deviation.

### 3.3. Identification of Bottleneck Nodes within 26SN

The identification of bottlenecks within 26SN was carried out using the Cytoscape (Cytoscape Consortuim, http://www.cytoscapeconsortium.org/) plugin CytoHubba. It implements the following algorithm for bottleneck calculation: let Ts be a shortest path tree rooted at node s. BN(v) = Σs∈V ps(v), where ps(v) = 1 if more than |V(Ts)|/4 paths from node s to other nodes in Ts meet at the vertex v; otherwise, ps(v) = 0 [35]. This parameter is a measure of the node centrality expressed as the number of shortest paths in which the node is involved, and which is related to the importance of nodes in controlling the information flux within directed networks [36].

### 3.4. Enrichment Analysis

To identify and predict new molecules possibly involved in proteasome network, we used Search Tool for the Retrieval of Interacting Genes/Proteins (STRING, http://string-db.org/newstring_cgi/show_input_page.pl?UserId = eNOo92_OQ_LS&sessionId = Cfz4mDP5ayne) [37]. It is a database including known and predicted protein interactions. They could be either direct (physical) or indirect (functional) associations, derived from different sources: genomic context, high-throughput experiments, conserved co-expression, and previous knowledge. From the data obtained using STRING, a new network was obtained by adopting a medium confidence score (0.400). Furthermore, to identify clusters of molecules within the network, we used a Markov cluster algorithm (MCL), setting the inflation parameter to 4.

## Figures and Tables

**Table 1:** Main topological parameters. The table lists the most relevant topological parameters in 26SN.

Parameter	Value
Connected components	1
Number of nodes	39
Number of links	47
Clustering coefficient	0.016
Networks diameter	8
Characteristic path length	3.121
Averaged number of neighbors	2.410
In degree	
γ	−2.231
r	0.983
R^2^	0.961
Out degree	
γ	−1.150
r	0.988
R^2^	0.934
**Most connected nodes**	**N° of links**
26S proteasome	13
PKA	9
Ca^2+^	7

For the parameter definitions, refer to Table 2 in Section 3.

**Table 2:** Main topological parameters assessed in this study.

Parameter	Definition
Connected components	The number of networks in which any two vertices are connected to each other by links, and which are connected to no additional vertices in the network.
Number of nodes	The total number of molecules involved.
Number of edges	The total number of interactions found.
Clustering coefficient	Calculated as *C*I = 2*n*I/*k*I(*k*I − 1), where *n*I is the number of links connecting the *k*I neighbors of node I to each other. It is a measure of how the nodes tend to form clusters.
Network diameter	The longest of all the calculated shortest paths in a network.
Characteristic path length	The expected distance between two connected nodes.
Averaged number of neighbors	The mean number of connections of each node.
Node degree	The number of interactions of each node.
Node degree distribution	Represents the probability that a selected node has *k* links.
	Exponent of node degree equation.
*R^2^*	Coefficient of determination of node degree vs. number of nodes, on logarithmized data.

## Supplementary Materials

The following are available online at https://www.mdpi.com/1422-0067/21/17/6256/s1.
